# Cell–Cell Communication at the Embryo Implantation Site of Mouse Uterus Revealed by Single-Cell Analysis

**DOI:** 10.3390/ijms22105177

**Published:** 2021-05-13

**Authors:** Yi Yang, Jia-Peng He, Ji-Long Liu

**Affiliations:** 1 College of Veterinary Medicine, Gansu Agricultural University, Lanzhou 730070, China; yang09yy@163.com; 2 Guangdong Laboratory for Lingnan Modern Agriculture, College of Veterinary Medicine, South China Agricultural University, Guangzhou 510642, China; stevencapeng@sina.com

**Keywords:** embryo implantation, mouse, single-cell RNA-seq, transcriptional changes

## Abstract

As a crucial step for human reproduction, embryo implantation is a low-efficiency process. Despite rapid advances in recent years, the molecular mechanism underlying embryo implantation remains poorly understood. Here, we used the mouse as an animal model and generated a single-cell transcriptomic atlas of embryo implantation sites. By analyzing inter-implantation sites of the uterus as control, we were able to identify global gene expression changes associated with embryo implantation in each cell type. Additionally, we predicted signaling interactions between uterine luminal epithelial cells and mural trophectoderm of blastocysts, which represent the key mechanism of embryo implantation. We also predicted signaling interactions between uterine epithelial-stromal crosstalk at implantation sites, which are crucial for post-implantation development. Our data provide a valuable resource for deciphering the molecular mechanism underlying embryo implantation.

## 1. Introduction

Embryo implantation is a crucial step for human reproduction. This is a low-efficiency process and the implantation failure rate per menstrual cycle is approximately 70% [1,2]. In assisted reproductive technologies, despite a high success rate of in vitro fertilization, implantation rate remains very low [3]. Additionally, an emerging concept is that early pregnancy loss and various pregnancy complications are rooted in suboptimal embryo implantation [4]. Therefore, it is imperative to understand the molecular mechanism of embryo implantation.

Due to ethical restrictions and experimental difficulties, in vivo analysis of embryo implantation heavily relies on mice. By using uterus-specific gene knockout mice, a number of genes have been proven to be required for embryo implantation [5]. Alternatively, several studies have analyzed global gene expression changes between implantation sites and inter-implantation sites of mouse uterus using high-throughput transcriptomic approaches, providing a useful candidate gene list for further study of embryo implantation [6,7,8]. The limitation of these studies is that the whole uterus is used. The uterus has a complex structure consisting of three layers, namely the endometrium, myometrium and perimetrium, and many cell types, including luminal and glandular epithelial cells, stromal cells, smooth muscle cells, endothelial cells and various immune cells. Thus, a whole uterus bulk RNA-seq approach is unable to accurately capture cell-type-specific gene expression changes. In addition to whole uteri, isolated uterine luminal epithelium from implantation sites and inter-implantation sites by enzymes [9] and laser-capture microdissection (LCM) [10] have been subjected to high-throughput transcriptomic analysis. It is undoubtedly true that the use of isolated uterine luminal epithelium was a better choice than the whole uterus. However, the limitation is that the contributions of cell types, other than luminal epithelium, and signaling crosstalk between different cell types were not considered.

With advances in single-cell RNA-seq techniques, it is now possible to analyze global gene expression profiles within highly heterogeneous tissues at the single-cell level [11]. In the present study, by using the-state-of-the-art single-cell RNA-seq approach, we resolved all cell types at implantation sites and inter-implantation sites of the mouse uterus on gestational day 5. Consequently, we were able to identify different expression changes associated with embryo implantation in all cell types. Additionally, we predicted that cell–cell communication is involved in embryo implantation. Our study provides a valuable resource for understanding the molecular mechanisms underlying embryo implantation in mice.

## 2. Results

### 2.1. Single-Cell Analysis of Embryo Implantation Site in Mouse Uterus

**Figure 1:**
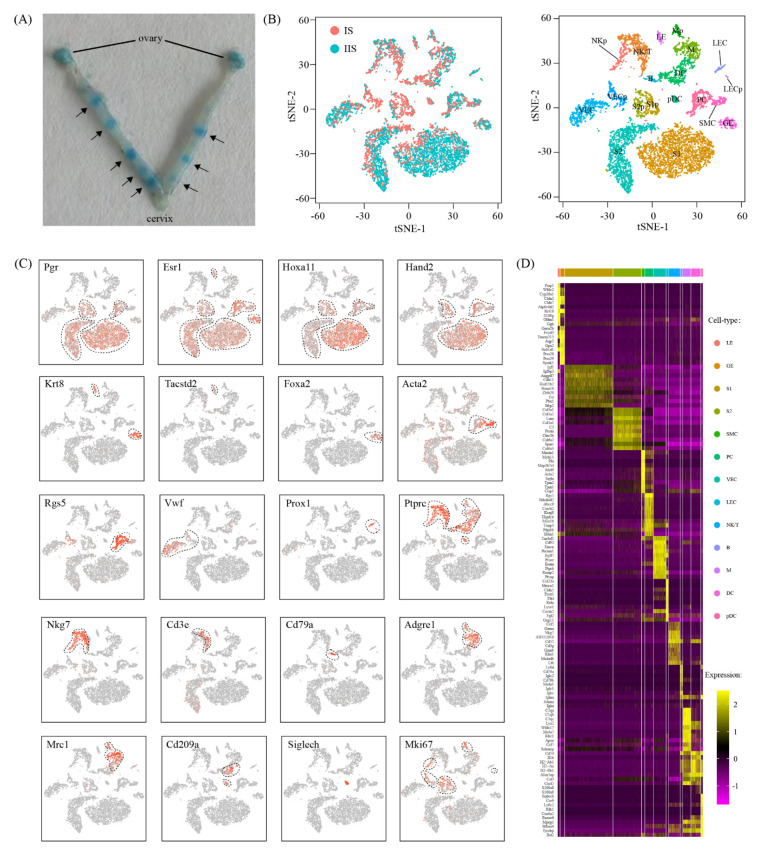
Single-cell transcriptome analysis of the implantation site in mouse uterus on gestational day 5: (**A**) A photograph of mouse uterus on gestational day 5. The position of embryo implantation sites was marked with an arrow. (**B**) The t-stochastic neighbor embedding (tSNE) representation of single-cell RNA-seq data obtained from implantation sites (IS) and inter-implantation sites (IIS). Single cells were grouped by cellular origin (left) and cell clusters (right). LE: luminal epithelial cells; GE: glandular epithelial cells; S1: superficial stromal cells; S2: deep stromal cells; S1p: proliferating superficial stromal cells; S2p: proliferating deep stromal cells; SMC: smooth muscle cells; PC: pericytes; VEC: vascular endothelial cells; VECp: proliferating vascular endothelial cells; LEC: lymphatic endothelial cells; VECp: proliferating lymphatic endothelial cells; M: macrophages; DC: dendritic cells; pDC: plasmacytoid dendritic cells; Mp: proliferating macrophages; NK/T: mixed natural killer cells and T cells; NKp: proliferating natural killer cells; B: B cells. (**C**) The expression pattern of canonical marker genes projected onto TSNE plots. Dashed lines denote the boundaries of the cell cluster of interest. (**D**) Heatmap of the top 10 gene expression signatures for each cell type.

Embryo implantation takes place on gestational day 5 in mice. The implantation site in the uterus can be visualized after an intravenous injection of Chicago Blue B dye solution (Figure 1A). In order to capture a cell-type resolved map of mouse embryo implantation, single-cell RNA-seq data were generated from the implantation site (IS) and the inter-implantation site (IIS, served as control) by using a 10x Genomics platform. The whole uterus, which consists of the endometrium, myometrium and perimetrium, was used. The blastocyst at IS was also kept. After quality control, a total of 8421 cells (2606 for IS and 5815 for IIS) were obtained. Unsupervised clustering analysis revealed 19 distinct cell clusters for IS and IIS combined (Figure 1B). Major cell types were defined using the expression of known cell-type-specific genes, with stromal cells expressing Hoxa11 [12], epithelial cells expressing Krt8 [13], smooth muscle cells expressing Acta2 [14], pericytes expressing Rgs5 [15], endothelial cells expressing Vwf or Prox1 [16] and immune cells expressing Ptprc [17] (Figure 1C).

We found four stromal cell clusters, S1, S1p, S2 and S2p. Cells in S1/S1p but not S2/S2p expressed high levels of Hand2, implying that S1/S1p were inner stromal cells and S2/S2p were outer stromal cells [18]. S1p and S2p expressed high level of Mki67, suggesting that S1p and S2p were a subset of proliferating S1 and S2 cells, respectively. There were two epithelial cell clusters, LE and GE. LE contained luminal epithelial cells expressing Tacstd2, and GE was composed of glandular epithelial cells expressing Foxa2 [19]. Only one smooth muscle cell cluster and one pericyte cluster were identified. Endothelial cells had four clusters: VEC and its proliferating subset VECp were vascular endothelial cells expressing Vwf, while LEC and its proliferating subset LECp were lymphatic endothelial cells expressing Prox1 [16]. The seven immune cell clusters were macrophages (M, Ptprc^+^Adgre1^+^) [20], a proliferating subset of macrophages (Mp, Ptprc^+^Adgre1^+^Mki67^+^), dendritic cells (DC, Ptprc^+^Cd209a^+^Siglech^−^) [20], plasmacytoid dendritic cells (pDC, Ptprc^+^Cd209a^+^Siglech^+^) [21], mixed natural killer cells and T cells (NK/T, Ptprc^+^Nkg7^+^ or Ptprc^+^Cd3e^+^) [22], a proliferating subset of natural killer cells (NKp, Ptprc^+^Nkg7^+^Cd3e^−^Mki67^+^) and B cells (B, Ptprc^+^Cd79a^+^) [22].

We next aimed to discover novel markers for each cell type. We selected genes that were expressed significantly higher in the cell type of interest than the other cell types by Wilcoxon rank sum test. A heatmap depicting the top 10 marker genes for each cell type is shown in Figure 1D. The complete lists of marker genes are presented in Supplementary Table S1.

### 2.2. Cell Type Proportion Changes at Implantation Site Compared to Inter-Implantation Site

**Figure 2:**
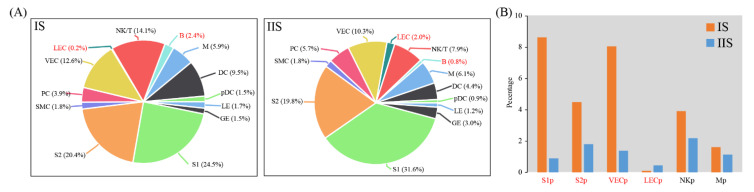
Cell population shifts in implantation sites compared to inter-implantation sites: (**A**) Pie plots showing the cell population change of 13 major cell types in implantation sites (IS) compared to inter-implantation sites (IIS) on gestational day 5. For each cell type, proliferating and non-proliferating cells were summed; (**B**) Bar plot showing cell population changes of proliferating cells. Cell types with fold change (based on percentage) > 2 and *p*-value (χ^2^ test) < 0.05 were labeled in red.

We investigated the abundance of each cell type at IS compared to IIS. For each cell type, proliferating and non-proliferating cells were summed. The χ^2^ test was employed to assess the significance of difference between two groups. By using the criteria of *p* < 0.05 and fold change > 2, the proportions of all cell types remained unchanged, except for LEC and B cells (Figure 2A). Of special interest, we investigated the abundance of proliferating cells. We found that the proportions of Mp and NKp were unchanged in IS compared to IIS. Notably, the proportion of LECp significantly decreased, whereas the proportions of S1p, S2p and VECp significantly increased (Figure 2B).

### 2.3. Cell-Type-Specific Transcriptional Changes Associated with Embryo Implantation

**Figure 3:**
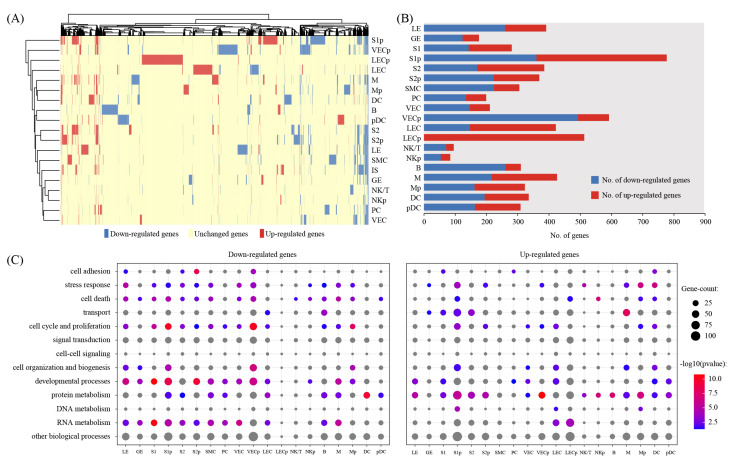
Gene expression changes in implantation sites compared to inter-implantation sites: (**A**) Heatmap showing the distribution of differentially expressed genes (logFC > 0.25 and *p* < 0.05) between implantation sites (IS) compared to inter-implantation sites (IIS) in each cell type; (**B**) the count of differentially expressed genes in each cell type; (**C**) gene ontology (GO) enrichment analysis of downregulated genes and upregulated genes, respectively. *p* < 0.05 was used as the significance cutoff. Non-significant hits (*p* ≥ 0.05) are depicted in gray.

We investigated the breadth of transcriptional changes in each cell type by performing differential gene expression analysis (Figure 3A). Using a logFC cutoff of 0.25 and a *p*-value cutoff of 0.05, we identified 391, 176, 281, 777, 385, 369, 305, 199, 211, 592, 422, 513, 95, 84, 310, 426, 323, 335 and 309 differentially expressed genes for LE, GE, S1, S1p, S2, S2p, SMC, PC, VEC, VECp, LEC, LECp, NK/T, NKp, B, M, Mp, DC and pDC, respectively (Figure 3B and Supplementary Table S2). We then explored the biological implications of differentially expressed genes using gene ontology (GO) analysis. A complete list of enriched GO terms is provided in Figure 3C. These data indicated that each cell type invokes distinct biological processes in order to participate in embryo implantation.

### 2.4. Inferring Cell–Cell Communication between Blastocyst and Uterus at the Implantation Site

**Figure 4:**
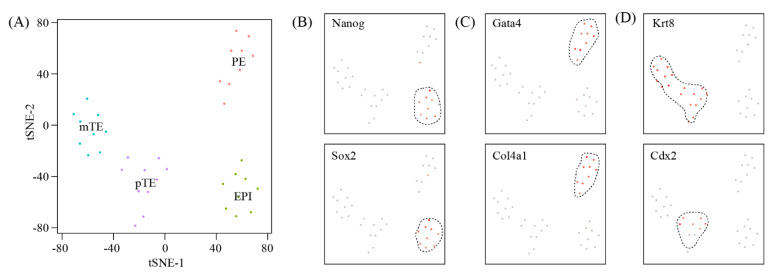
A single-cell atlas of E4.5 embryos: (**A**) TSNE clustering of single cells from mouse E4.5 blastocysts which were collected from GD5 uteri from a previous study. EPI: epiblast; PE: primitive endoderm; mTE: mural trophectoderm; pTE: polar trophectoderm. (**B**–**D**) TSNE plots showing the expression pattern of canonical marker genes for EPI (**B**), PE (**C**) and mTE/pTE (**D**). Dashed lines show the boundaries of the specific cell clusters.

The cell–cell communication between blastocyst and uterus represents the key mechanism of embryo implantation. Due to the relatively small number of blastocyst cells at the embryo implantation site, we did not find any blastocyst-related cell clusters in our single-cell RNA-seq data (data not shown). Alternatively, we re-analyzed a published single-cell RNA-seq dataset on mouse E4.5 blastocysts [23] (Figure 4A). E4.5 is equivalent to gestational day 5 in our study. By using canonical marker genes, four major cell types were identified: epiblast (EPI) (Figure 4B), primitive endoderm (PE) (Figure 4C), polar trophectoderm (pTE) and mural trophectoderm (mTE) (Figure 4D).

**Figure 5:**
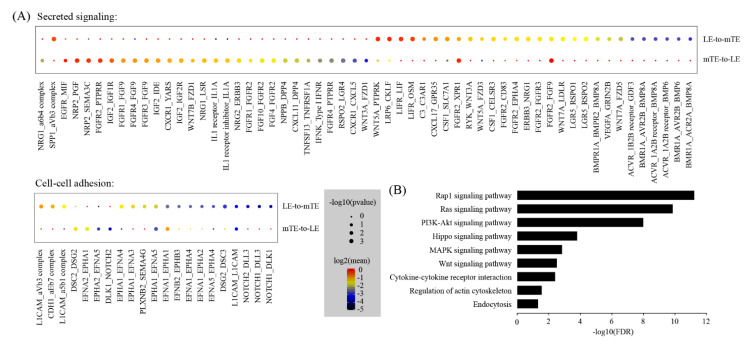
Cell–cell communication between mTE and LE: (**A**) Dot plot showing selected ligand–receptor interactions underlying mTE-LE crosstalk. *p*-values are indicated by circle size and means of the average expression level of interacting molecule are indicated by color; (**B**) KEGG Pathway enrichment analysis of ligand–receptor pairs by using the DAVID online tools.

During mouse embryo implantation, the blastocysts attach to the LE of the uterus in the way of mTE. Thus, we used CellPhoneDB (https://www.cellphonedb.org/ (accessed 22 April 2021)) to predict the ligand–receptor interactions between LE and mTE. We found a total of 78 ligand–receptor interaction pairs (Figure 5A). Based on pathway analysis, these ligand–receptor interactions were enriched among Rap1 signaling pathway (FDR = 6.1 × 10^−12^), Ras signaling pathway (FDR = 1.4 × 10^−10^), PI3K-Akt signaling pathway (FDR = 1.0 × 10^−8^), Hippo signaling pathway (FDR = 1.6 × 10^−4^), MAPK signaling pathway (FDR = 0.0014), Wnt signaling pathway (FDR = 0.0031), cytokine–cytokine receptor interaction (FDR = 0.0039), regulation of actin cytoskeleton (FDR = 0.028) and endocytosis (FDR = 0.049) (Figure 5B).

### 2.5. Inferring Uterine Epithelial-Stromal Crosstalk at the Implantation Site

**Figure 6:**
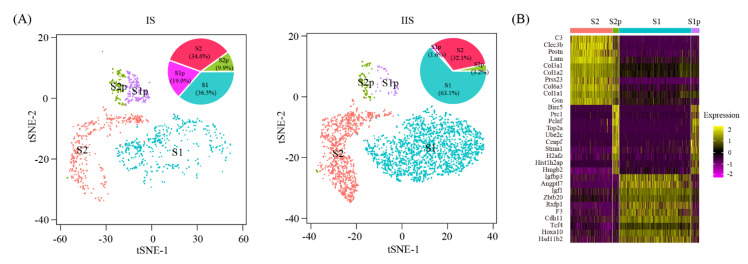
Sub-clusters of stromal cells: (**A**) TSNE plot showing four clusters of stromal cells. Relative cell type abundance was shown as insets; (**B**) heatmap of top 10 gene expression signatures for each cell type.

It has been well established that uterine epithelial-stromal crosstalk is important for embryo implantation [24]. In our single-cell RNA-seq data, we identified four clusters of stromal cells: S1, S1p, S2 and S2p. Their relative abundance was 36.5%, 19.0%, 34.6%, 9.9% at IS, and 63.1%, 1.6%, 32.1%, 3.2% at IIS (Figure 6A). By analyzing the gene signature for each cell cluster, we confirmed that S1p was related to S1 and S2p was related to S2. S1p and S2p were proliferating subsets of S1 and S2, respectively (Figure 6B).

**Figure 7:**
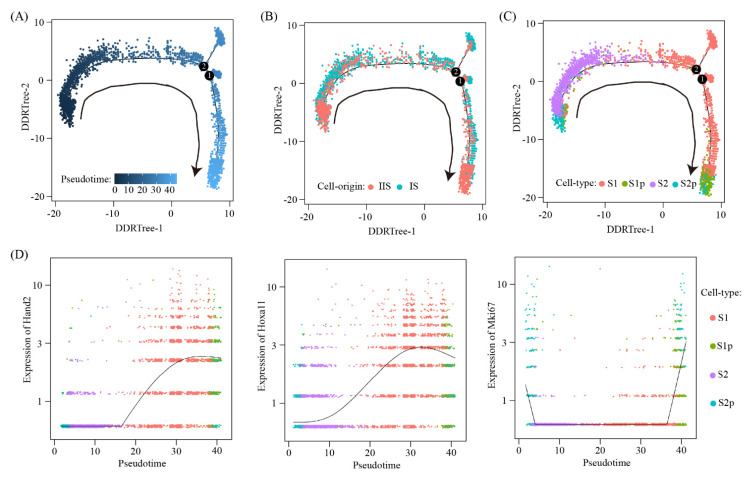
Pseudotime ordering of stromal cells: (**A**–**C**) The distribution of pseudotime (**A**), cell origin (**B**) and cell type (**C**) across the reconstructed trajectory. The numbers inside the black circles represent the different cell status numbers identified in the trajectory analysis; (**D**) line plots showing gene expression dynamics of Hand2, Hoxa11 and Mki67 in the pseudotime order.

To further reveal the relationship between these four stromal cell clusters, pseudotime trajectory analysis was conducted. Cells were arranged in a pseudotime manner with a pedigree reconstruction algorithm for biological processes based on transcriptional similarity. We found that all stromal cell clusters formed a continuous trajectory (Figure 7A–C). We mapped three marker genes, namely Hand2, Hoxa11 and Mki67, on the trajectory (Figure 7D). Our results suggested a S2p → S2 → S1 → S1p trajectory: (1) S2p was the origin of all stromal cells; (2) S2 was able to transit to S1; and (3) S1p was derived from S1.

**Figure 8:**
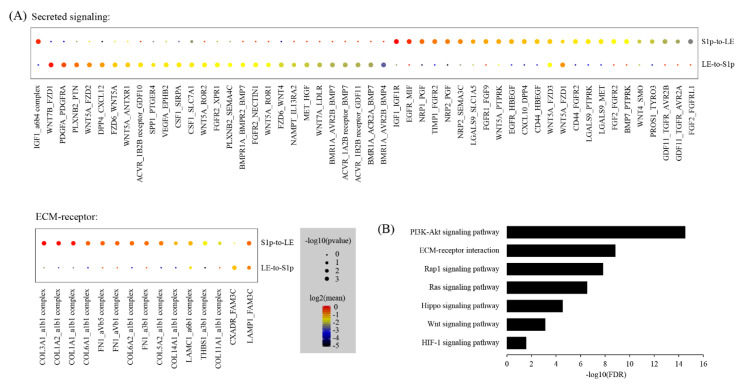
Cell–cell communication between LE and S1p: (**A**) dot plot showing selected ligand–receptor interactions underlying LE-S1p crosstalk; (**B**) KEGG Pathway enrichment analysis of ligand–receptor pairs.

Upon embryo implantation, stromal cells proximally surrounding the implantation chamber (i.e., S1p cells) exhibit proliferation and spreading. These cells differentiate and form the primary decidual zone (PDZ) on the afternoon of gestational day 5 [25,26]. Thus, the interaction between LE and S1p represents the uterine epithelial-stromal crosstalk. By using CellPhoneDB, we predicted the ligand–receptor interactions between LE and S1p. We identified a total of 77 ligand–receptor interaction pairs (Figure 8A). Based on pathway analysis, these ligand–receptor interactions were enriched among the PI3K-Akt signaling pathway (FDR = 2.9 × 10^−15^), ECM-receptor interaction (FDR = 1.4 × 10^−9^), Rap1 signaling pathway (FDR = 1.4 × 10^−8^), Ras signaling pathway (FDR = 2.9 × 10^−7^), Hippo signaling pathway (FDR = 2.8 × 10^−5^), Wnt signaling pathway (FDR = 7.3 × 10^−4^) and HIF−1 signaling pathway (FDR = 0.026) (Figure 8B).

## 3. Discussion

The mouse is widely used as an animal model for investigating human embryo implantation. In this study, by profiling the single-cell transcriptome for 8421 cells using the 10x Genomics approach, we revealed 19 distinct cell clusters, including four stromal cell clusters, two epithelial cell clusters, one smooth muscle cell cluster, one pericyte cluster, four endothelial cell clusters and seven immune cell clusters at the mouse embryo implantation site. To the best of our knowledge, the present study is the first to highlight the transcriptome landscape of embryo implantation at single-cell resolution.

In our single-cell RNA-seq data, the cell type composition for the implantation site of the uterus is 3.2% epithelial cells, 44.9% stromal cells, 1.8% smooth muscle cells, 3.9% pericytes, 12.8% endothelial cells and 33.4% immune cells, while the cell type composition for the inter-implantation site of the uterus is 4.2% epithelial cells, 41.4% stromal cells, 1.8% smooth muscle cells, 5.7% pericytes, 12.3% endothelial cells and 20.1% immune cells. By using the criteria of *p* < 0.05 and fold change >2, the proportions of all cell types were unchanged except for LEC and B cells. In fact, LEC cells and B cells are minor cell types (<5%) in the uterus, and thus the estimated percentages for these two cell types might be inaccurate. These results were in line with the fact that there are only minimal histological changes at the implantation site compared to the inter-implantation site on gestational day 5 [6].

We investigated the breadth of transcriptional changes for each cell type by performing differential gene expression analysis. S1p cells have the largest number of differentially expressed genes. In this study, we identified four stromal cell clusters: S1, S1p, S2 and S2p. S1 and its proliferating subset S1p were superficial stromal cells expressing Hand2, while S2 and its proliferating subset S2p were deep stromal cells which were negative for Hand2. Upon embryo implantation, stromal cells proximally surrounding the implantation chamber (i.e., S1p cells) exhibit proliferation and spreading. These cells differentiate and form the primary decidual zone (PDZ) on the afternoon of gestational day 5 [25,26]. Gene ontology analysis showed that stress response, cell death, transport, cell cycle and proliferation, cell organization and biogenesis, developmental processes, protein metabolism, DNA metabolism and RNA metabolism were significantly enriched among differentially expressed genes. We were also interested in genes differentially expressed in LE. Blastocyst attachment to the uterine LE occurs at midnight of gestational day 4 and a firm connection between blastocyst attachment to the uterine LE is established on the morning of gestational day 5 [27]. We identified 391 differentially expressed genes in LE, of which 132 genes were upregulated and 259 genes were downregulated in IS compared to IIS. Gene ontology analysis revealed that cell adhesion, stress response, cell death, cell cycle and proliferation, cell organization and biogenesis, developmental processes, protein metabolism and RNA metabolism were significantly enriched among differentially expressed genes. Considering spatial relationships between cell types at the implantation site, LE and S1p cells are likely the top two contributors to embryo implantation in the uterus. Nevertheless, we would like to note that immune cells (especially M and DC) [28], as well as GE [29], may also largely contribute to embryo implantation according to the literature.

Previously, the E4.5 blastocysts obtained from GD5 uteri have been subjected to single-cell RNA-seq [23]. Through data integration, we inferred cell–cell communication between E4.5 blastocysts and the implantation site of GD5 uteri by their expression of ligand–receptor pairs. We were particularly interested in the interactions between mTE from the blastocyst and LE from the uterus, as physical contact between these two cell types represents the key mechanism of embryo implantation. Notably, LE cells expressed FGF9, while the corresponding receptors (FGFR1, FGFR3 and FGFR4) were expressed in mTE. On the other hand, mTE cells expressed FGF4 and FGF10, while the corresponding receptor FGFR2 was expressed in mTE. These data highlight the importance of FGF signaling in embryo implantation. The FGF signaling is part of the Rap1 signaling pathway, Ras signaling pathway, PI3K-Akt signaling pathway, MAPK signaling pathway and cytokine-cytokine receptor interaction. Additionally, we found that Wnt signaling pathway and Hippo signaling pathway might also play a significant role in LE-mTE interaction. Previous studies have shown that uterine epithelial-stromal crosstalk is crucial for embryo implantation as well as post-implantation development of the uterus [24]. In this study, we narrowed down uterine epithelial-stromal crosstalk to the interaction between LE and S1p cells. We found that FGF and Wnt signaling also play an important role in LE-S1p interaction. Although the interactions between LE/S1p and immune cells might also contribute to embryo implantation, they were not pursued in this study.

In conclusion, this study provided a comprehensive single-cell transcriptome atlas for the mouse embryo implantation site. Our data present a valuable resource for deciphering the molecular mechanism underlying embryo implantation.

## 4. Materials and Methods

### 4.1. Sample Collection

Adult SPF-grade CD-1 mice were used in this study. All mice were caged under light-controlled conditions with 14 h/10 h light/dark cycles and free access to regular food and water. Female mice were mated with fertile males and the mating was confirmed the next morning by the presence of a vaginal plug. The day of the vaginal plug was denoted as gestation day 1 (GD1). On the morning of GD5, embryo implantation positions along the uterus were identified after an intravenous injection of Chicago Blue B dye solution. The whole uterus segments for implantation sites (IS) and inter-implantation sites (IIS) were collected separately. All animal procedures were approved by the Institutional Animal Care and Use Committee of South China Agricultural University (Guangzhou, China).

### 4.2. Single-Cell Dissociation of Mouse Uterus

Whole uterus segments were incubated in the dissociation buffer containing 2 mg/mL Collagenase II (#C6885, Sigma-Aldrich, St. Louis, MO, USA), 10 mg/mL Dispase II (#354235, Corning, Corning, NY, USA) and 50,000 U/mL DNase I (#DN25, Sigma-Aldrich, St. Louis, MO, USA) for up to 30 min at 37 °C in a shaking incubator. The digestion progress was checked every 5 min with a microscope until single-cell suspension was achieved. The single-cell suspension was then passed through a 40-μm cell strainer to remove undigested tissues. Cells were spun down at 250× *g* at 4 °C for 4 min and the pelleted cells were washed using centrifugation. In order to measure cell viability, cells were strained with AO/PI solution (#CS2-0106, Nexcelom Bioscience, Lawrence, MA, USA) and counted using a Cellometer Auto 2000 instrument (#SD-100, Nexcelom Bioscience, Lawrence, MA, USA). The single-cell suspension was carried forward to single-cell RNA-seq only if the cell viability was >80% and the percentage of cell clumps was <10%.

### 4.3. Single-Cell RNA-Seq Library Preparation and Sequencing

The final concentration of single-cell suspension was adjusted to 1000 cells/μL and a volume of 15 µL was loaded into one channel of the Chromium^TM^ Single Cell B Chip (#1000073, 10x Genomics, Pleasanton, CA, USA), aiming at recovering 8000–10,000 cells. The Chromium Single Cell 3′ Library and Gel Bead Kit v3 (#1000075, 10x Genomics) was used for single-cell bar-coding, cDNA synthesis and library preparation, following the manufacturer′s instructions provided as the Single Cell 3′ Reagent Kits User Guide Version 3. Library sequencing was performed on a NovaSeq 6000 system (Illumina, San Diego, CA, USA) configured with the paired-end 150-bp protocol at a sequencing depth of approximately 400 million reads.

### 4.4. Single-Cell RNA-Seq Data Analysis

Raw data of bcl files from the NovaSeq 6000 system were converted to fastq files using the bcl2fastq2 tool v2.19.0.316 (Illumina, San Diego, CA, USA). These fastq files were aligned to the 10 mm mouse reference genome by using the CellRanger software v3.0.1 (10x Genomics, Pleasanton, CA, USA). The resulting gene count matrix was processed with the R package Seurat v3.1.3 [30]. Cells with fewer than 200 or greater than 6000 unique genes, as well as cells with greater than 25% mitochondrial counts, were excluded. Meanwhile, genes expressed in fewer than 3 cells were removed. Following data filtering, the clean gene count matrix was normalized and scaled by using NormalizeData and ScaleData, respectively. The top 2000 highest variable genes were used for the principal component analysis (PCA) and the optimal number of PCA components was determined by the JackStraw procedure. Single cells were clustered by the graph-based algorithm in PCA space and visualized using the t-distributed stochastic neighbor embedding (tSNE) dimensional reduction technique. The cell type label for each cell cluster was manually assigned based on canonical cell markers. The FindAllMarkers function was used to identify novel marker genes for each cluster with a minimum of 20% of cells expressing the gene within the cluster and a minimum logFC threshold of 0.25. In order to find differentially expressed genes in the same cell type between pre-receptive uteri and receptive uteri, the FindMarkers function in Seurat was used with min.pct being set to 0.20 and min.logfc being set to 0.25.

### 4.5. Gene Ontology Analysis

Gene ontology (GO) analysis was performed as described previously [31]. GO terms were grouped according to the biological process category in the Mouse Genome Informatics (MGI) GOslim database [32]. To test for enrichment, a hypergeometric test was conducted and *p* < 0.05 was used as the significance threshold to identify enriched GO terms.

### 4.6. Pathway Enrichment Analysis

Pathway enrichment analysis was conducted by using the DAVID online tools 6.8 [33]. The significance threshold for FDR was set at 0.05.

### 4.7. Cell–Cell Communication Analysis

The single-cell RNA-seq data for mouse E4.5 blastocysts were downloaded from the GEO database (GSE63266) [23] and re-analyzed with the same pipeline as described above. E4.5 is equivalent to GD5. In order to analyze cell–cell communication between blastocysts and uteri, we merged E4.5 blastocyst data and uterus IS data into a single Seurat object, from which a meta file as well as a count file was generated. These 2 files were used as input for the CellphoneDB software v2.1.4 [34] to infer cell–cell communication based on ligand–receptor interactions with default parameters. *p* < 0.05 were considered statistically significant.

### 4.8. Pseudotime Analysis

The Monocle2 package v2.18.0 was used for pseudotime analysis [35]. The count data and meta data were exported from the Seurat object and then imported into the CellDataSet object in Monocle2. Feature genes were selected by using the differentialGeneTest function. After reducing dimensions using the DDRTree algorithm, the orderCells function was used to infer the trajectory with default parameters. The reconstructed trajectory was visualized by the plot_cell_trajectory function. The plot_genes_in_pseudotime function was used to map gene expression dynamics.

## Data Availability

The data presented in this study are available on request from the corresponding author.

## Supplementary Materials

The following are available online at https://www.mdpi.com/article/10.3390/ijms22105177/s1.
